# Hippocampal gene expression dysregulation of Klotho, nuclear factor kappa B and tumor necrosis factor in temporal lobe epilepsy patients

**DOI:** 10.1186/1742-2094-10-53

**Published:** 2013-05-01

**Authors:** Marcelo Ananias Teocchi, Ana Érika Dias Ferreira, Evandro Pinto da Luz de Oliveira, Helder Tedeschi, Lília D’Souza-Li

**Affiliations:** 1 Laboratory of Pediatric Endocrinology, Center for Investigation in Pediatrics, University of Campinas, PO Box 6111, Campinas, SP, 13083-970, Brazil; 2 Department of Neurology, Faculty of Medical Sciences, University of Campinas, PO Box 6111, Campinas, SP, 13083-970, Brazil

**Keywords:** Seizures, Hippocampal sclerosis, Neuroinflammation, TNF, KL, NFKB1, GFAP, Reference genes, Calcium homeostasis

## Abstract

**Background:**

Previous research in animal seizure models indicates that the pleiotropic cytokine TNF is an important effector/mediator of neuroinflammation and cell death. Recently, it has been demonstrated that TNF downregulates Klotho (KL) through the nuclear factor kappa B (NFkB) system in animal models of chronic kidney disease and colitis. KL function in the brain is unclear, although Klotho knockout (*Kl*^*−/−*^) mice exhibit neural degeneration and a reduction of hippocampal synapses. Our aim was to verify if the triad *KL*-*NFKB1*-*TNF* is also dysregulated in temporal lobe epilepsy associated with hippocampal sclerosis (TLE(HS)) patients.

**Findings:**

We evaluated *TNF*, *NFKB1* and *KL* relative mRNA expression levels by reverse transcription quantitative PCR (RT-qPCR) in resected hippocampal tissue samples from 14 TLE(HS) patients and compared them to five *post mortem* controls. Four reference genes were used: *GAPDH*, *HPRT1*, *ENO2* and *TBP*. We found that *TNF* expression was dramatically upregulated in TLE(HS) patients (*P* <0.005). *NFKB1* expression was also increased (*P* <0.03) while *KL* was significantly downregulated (*P* <0.03) in TLE(HS) patients. Hippocampal *KL* expression had an inverse correlation with *NFKB1* and *TNF*.

**Conclusions:**

Our data suggest that, similar to other inflammatory diseases, TNF downregulates KL through NFkB in TLE(HS) patients. The remarkable *TNF* upregulation in patients is a strong indication of hippocampal chronic inflammation. Our finding of hippocampal *KL* downregulation has wide implications not only for TLE(HS) but also for other neuronal disorders related to neurodegeneration associated with inflammation.

## Findings

### Introduction

Temporal lobe epilepsy (TLE) is the most treatment resistant (refractory) partial epilepsy and only 20% of patients achieve seizure control with antiepileptic drugs (AEDs) [1]. Hippocampal sclerosis (HS) is the main pathological finding observed in excised tissue from TLE patients treated with amygdalohippocampectomy. Only 10% of TLE patients with HS, who are treated with AEDs, become seizure-free which emphasizes the importance of HS as a prognostic factor [1]. HS is characterized by an abnormal increase in the number of astrocytes associated with the destruction of nearby neurons (astrogliosis) [2]. One of the primary events in seizure-induced cell death in the hippocampus is the excessive release of glutamate with consequent overload in intracellular calcium influx [3]. Within the neuronal cells, sophisticated homeostatic mechanisms control calcium levels. Altered Ca^2+^ sensitivity or defective Ca^2+^ regulation appear to be involved in the aging process, contributing to the progressive neurodegeneration in Alzheimer’s disease and the intensified susceptibility to cell death after a seizure or stroke [3,4].

Klotho (KL), originally identified as an anti-aging protein, is involved in multiple functions in many systems and acts as an important calciophosphoregulatory hormone [5]. *KL* mRNA is expressed only in limited organs, that is the brain, kidney, reproductive organs, pituitary gland and parathyroid glands [6,7]. Its cerebral function is unclear, however Klotho knockout (*Kl*^*−/−*^) mice exhibit a phenotype resembling human aging, showing neural degeneration and a reduction of synapses in the hippocampus [6]. In addition, KL regulates the cellular lifespan of human cells by repressing the pro-apoptotic tumor protein p53 (TP53) [8], which regulates a number of apoptosis-related genes.

Important components and features of medial TLE, such as hippocampal intracellular calcium imbalance, neurodegeneration, hippocampal atrophy and cognitive decline, led us to question whether KL would also be downregulated in temporal lobe epilepsy associated with hippocampal sclerosis (TLE(HS)) patients. Furthermore, several studies assert that inflammation has a crucial role in epileptogenesis [9] and an increasing body of evidence connects astrogliosis to neuroinflammation [10]. In epilepsy, the pleiotropic cytokine TNF is indicated as being an important effector/mediator of neuroinflammation and cell death [9,11-15]. Interestingly, it has been demonstrated that TNF downregulates KL through the transcription nuclear factor kappa B (NFkB) in animal models of chronic kidney disease and colitis [16,17]. Since inflammation and neurodegeneration seem to be connected in HS, our objective was to verify if the triad *KL*-*NFKB1*-*TNF* is also dysregulated in TLE(HS).

## Methods

Ethical approval was certified by the “Comitê de Ética da Faculdade de Ciências Médicas da Unicamp (CEP n 470/2003)”.

### Patients, *post mortem* controls and tissues

Electroencephalogram (EEG) video monitoring/telemetry was performed on all patients to confirm the onset of seizure in the medial temporal lobe. Dual pathologies or multifocal epilepsies were not identified. Hippocampal atrophy was detected by magnetic resonance imaging (MRI) in all patients.

Each patient signed an informed consent agreement to allow scientific use of the tissue. All procedures were carried out with the approval of the local research ethics committee, and in compliance with institutional guidelines and relevant laws.

Fourteen TLE(HS) patients had the amygdalohippocampectomy procedure performed for therapeutic reasons (Table 1). Hippocampal tissue samples from all 14 patients were immediately collected and divided into two parts. One portion was immediately snap-frozen in liquid nitrogen and stored at −80°C until RNA isolation occurred. The second portion was fixed for histopathological analysis and HS was confirmed in all of them.

**Table 1:** Clinical and demographic features of TLE(HS) patients

**Patients**	**Gender**	**Age (years)**	**Side of HS**	**Duration (years)**	**Last seizure (days before surgery)**	**TLE form**	**AED during surgery**
TLE 01	F	34.6	L	28.6	nd	Familial	PHT
TLE 02	F	29.1	L	22.1	2	Sporadic	CBZ
TLE 03	F	23.8	R	22.3	7	Sporadic	CBZ
TLE 04	M	42.8	R	41.2	3	Sporadic	CBZ
TLE 05	M	41.2	L	34.2	5	Sporadic	CBZ
TLE 06	M	50.8	R	48.8	7	Sporadic	CBZ
TLE 07	M	12.7	L	9.7	3	Sporadic	CBZ, PHT
TLE 08	F	43.8	B(L)^a^	41.8	nd	Sporadic	CBZ, LTG
TLE 09	F	58.2	R	57.7	nd	Sporadic	OXC, VPA
TLE 10	F	54.9	L	50.9	nd	Sporadic	CBZ
TLE 11	F	32.1	L	31.6	nd	Familial	CBZ
TLE 12	F	38.3	L	37.0	2	Familial	OXC
TLE 13	F	54.1	L	53.3	nd	Sporadic	OXC, PHT
TLE 14	M	34.4	R	34.2	nd	Familial	OXC

^a^The hippocampal side was more affected when bilateral. When the patient’s last seizure was ‘nd’, the seizure most likely occurred over 7 days prior to surgery. TLE(HS), temporal lobe epilepsy associated with hippocampal sclerosis; M, male; F, female; B, bilateral; R, right; L, left; nd, not determined; AED, antiepileptic drug; CBZ, carbamazepine; PHT, phenytoin; OXC, oxcarbazepine; LTG, lamotrigine; VPA, valproate.

Five *post mortem* control hippocampal tissue samples (one female, four males; 28.2 ± 13.1 years; range from 19 to 50 years old) were provided by the Instituto Médico Legal (IML) de Campinas. Despite some traumatic deaths, no neurological abnormalities were detected. Subjects passed away unexpectedly and instantly, which minimizes the occurrence and progression of neuroinflammation. The *post mortem* delay averaged 7.8 hours (range from 6 to 9 hours).

### RNA extraction and reverse transcription quantitative PCR (RT-qPCR)

To extract total RNA, 1 ml of TRIzol Reagent (Life Technologies, Foster City, CA, USA) was added per 75 mg of frozen tissue samples, homogenized and then further processed according to the manufacturer’s instructions. The RNA integrity number (RIN) mean in both the control and patient groups was similar: 6.68 ± 0.9441 (n = 5) and 6.155 ± 0.2484 (n = 11), respectively. Due to the fact that the RNA was unavailable, the RIN was not evaluated for three patient samples: TLE 03, TLE 11 and TLE 13. Subsequently, 1 μg of total RNA of each sample was reverse transcribed into cDNA using 200 U of Superscript III Reverse Transcriptase (Life Technologies) and 3 μg of Random Primers (Life Technologies) according to the manufacturer’s instructions.

Sterilized and filtered DEPC-treated water was used in all cDNA synthesis reactions. Complementary DNA samples derived from the investigated genes were detected using an ABI PRISM 7500 Sequence Detection System (Life Technologies) and TaqMan Gene Expression Assays (Life Technologies): 5′-FAM-labeled probes and corresponding primer pairs (Table 2). Gene names are in accordance with the approved symbol from the HUGO Gene Nomenclature Committee (HGNC) database. To select the reference genes (endogenous controls), the study of Wierschke *et al.* on human epileptogenic tissues was considered [18]. Among 12 candidate genes, the algorithm NormFinder indicated hypoxanthine phosphoribosyltransferase 1 (*HPRT1*), enolase 2 (gamma, neuronal) (*ENO2*) and TATA box binding protein (*TBP*) as good normalization factors, since as single genes their expression levels were among the five most stable. Glyceraldehyde-3-phosphate dehydrogenase (*GAPDH*), a very often used reference gene, showed a relatively unstable expression. However, to reinforce our results, we opted to test all four reference genes. Glial fibrillary acidic protein (*GFAP*) was used as an upregulation control [18-20]. Each qPCR was run as triplicates with 10 ng cDNA sample in 6.25 μl TaqMan Gene Expression Master Mix (Life Technologies), 0.625 μl of the respective probe/primer mix, and 0.625 μl purified and deionized H_2_O.

**Table 2:** Genes and gene expression assays analyzed in this study

**Gene symbol**	**Name**	**Task**	**TaqMan Assay number**	**Amplicon (bp)**	**Slope**	**R**^**2**^	**Efficiency**
*ENO2*	Enolase 2 (gamma, neuronal)	Reference gene	Hs00157360_m1	77	−3.356	0.994	98.61
*GAPDH*	Glyceraldehyde-3-phosphate dehydrogenase	Reference gene	4333764 F	122	−3.313	0.996	100.36
*GFAP*	Glial fibrillary acidic protein	Upregulation marker	Hs00909236_m1	59	−3.414	0.999	96.29
*HPRT1*	Hypoxanthine phosphoribosyltransferase 1	Reference gene	4333768 F	100	−3.306	0.988	100.67
*KL*	Klotho	Target	Hs00183100_m1	74	−3.396	0.998	97.02
*NFKB1*	Nuclear factor of kappa light polypeptide gene enhancer in B-cells 1	Target	Hs00765730_m1	66	−3.388	0.997	97.30
*TBP*	TATA box binding protein	Reference gene	Hs99999910_m1	127	−3.407	0.993	96.57
*TNF*	Tumor necrosis factor	Target	Hs99999043_m1	85	−3.266	0.991	102.39

The amplification of all samples was of the same efficiency for precise quantification of reverse transcription quantitative PCR (RT-qPCR) data. Serial fivefold dilutions, starting with 250 ng of cDNA from the RNA quantification, of a cDNA solution pooled from the control group was used. The mean C_T_ values, measured in triplicates, versus the log10 of the dilution was plotted. The values from linear regressions applied to these plots were also presented (not shown). The amplification efficiencies (E = 10^(−1/slope)^) were close to 1.0 (100%).

### Data analysis

Relative gene expression quantification data was generated and analyzed using the 7500 Software version 2.0.5 (Life Technologies). One of the *post mortem* control samples was randomly chosen as the benchmark and the quantification data from the other samples, including controls and patients, was evaluated according to this reference sample, which always had a relative quantification of 1.0. This allowed the two groups (*post mortem* controls and TLE(HS) patients) to be statistically compared.

The GraphPad Prism 5 software version 5.04 for Windows was used for the statistical analysis (San Diego, CA, USA; http://www.graphpad.com). The Mann–Whitney *U* test was used for comparison between data from the control group (n = 5) versus the patient group (n = 14). All comparison data are presented as mean and SD. Correlation among the target genes was performed by the Spearman’s rank correlation (Spearman’s R). The reference sample was excluded from correlation tests and differences of *P* <0.05 were considered significant.

## Results and discussion

The quality of the RNA and the reliability of reference genes to quantify gene expression in surgical tissue are crucial in interpreting epilepsy-related changes of gene expression [18,21]. The similarity in the RIN average between TLE patients and the control group (6.68 ± 0.9441 and 6.155 ± 0.2484, respectively) reinforces our results. *Post mortem* delay has always been considered a major factor when interpreting the accuracy of results from human tissue studies; however, this may not be a factor for human brain RNA [21]. Durrenberger *et al.* concluded that *post mortem* delay caused only a minor deterioration and had no effect on RNA quality. The same results were found in previous research mentioned in their report [21]. We worked with a *post mortem* delay of between 6 to 9 hours, which would not seem to contradict the findings of the Durrenberger study.

**Figure 1:**
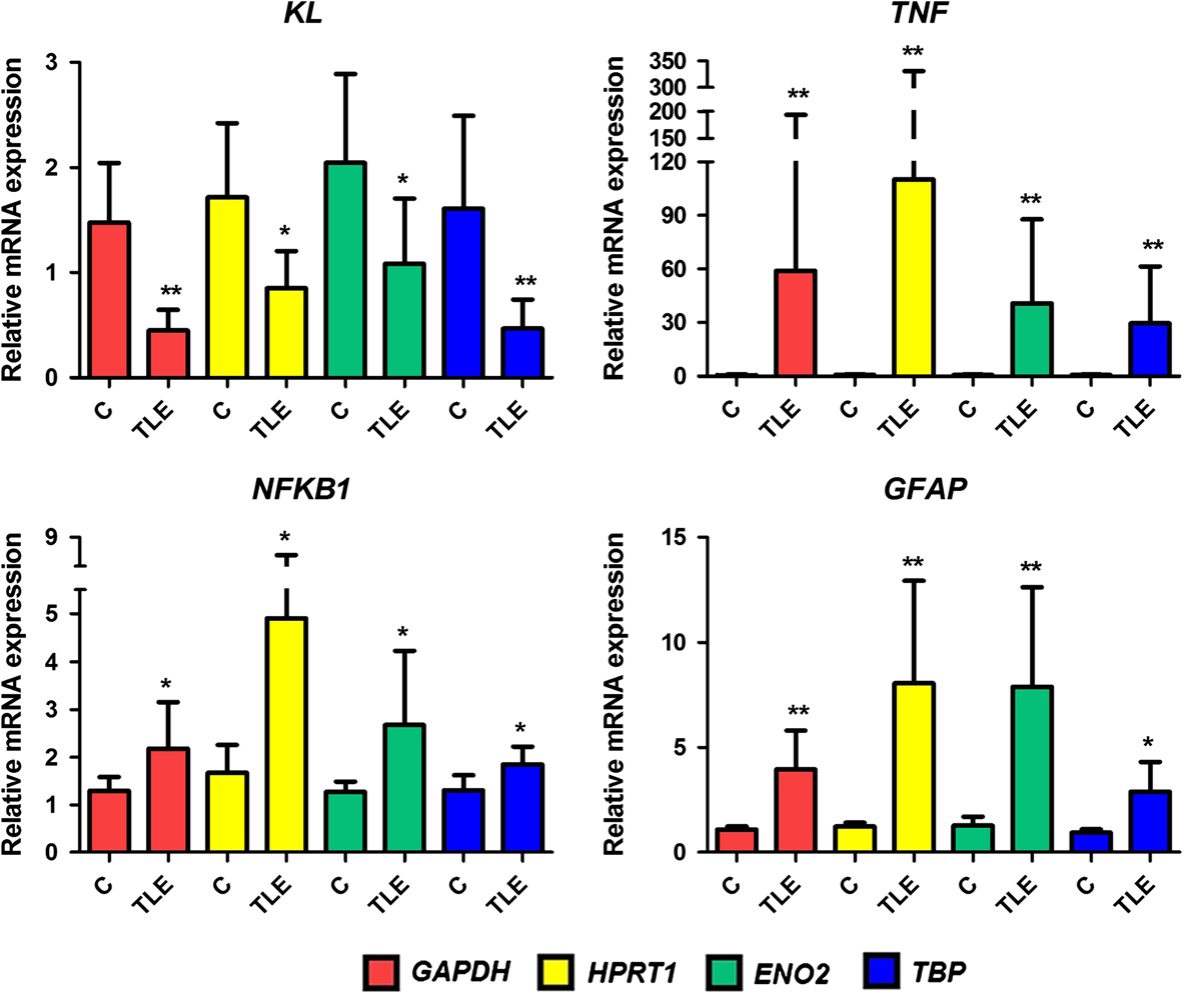
**Hippocampal relative gene expression of *KL*, *NFKB1*, *TNF *and *GFAP *in the TLE(HS) patient group versus the *post mortem *control group.** Different colors represent the four reference genes used: *GAPDH* (red), *HPRT1* (yellow), *ENO2* (green) and *TBP* (blue). For the three target genes (*KL*, *NFKB1* and *TNF*) and the upregulation marker (*GFAP*), four independent comparisons were performed, since each reference gene was considered as an independent variable. The y-axis represents the quantitative data of the relative mRNA expression of the target molecules in sclerotic hippocampal tissues of the TLE(HS) patient group (n = 14) compared with the *post mortem* control group (n = 5). All data are presented as mean and SD. Mann–Whitney U tests were used for comparisons between groups. *P <0.05, **P <0.01. TLE(HS), temporal lobe epilepsy associated with hippocampal sclerosis.

**Figure 2:**
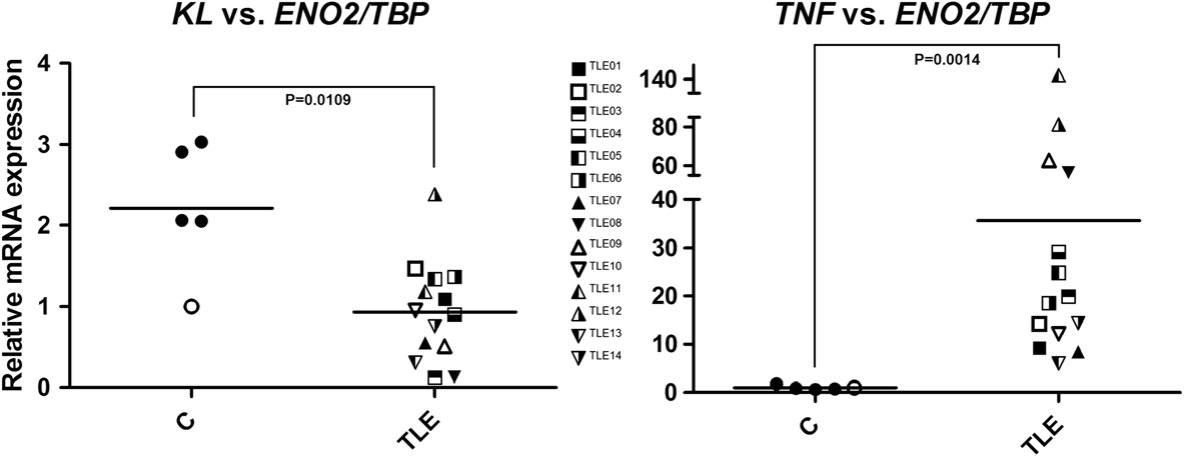
**Dispersion of the relative gene expression of *KL *and *TNF *in the hippocampus of TLE(HS) patients and *post mortem *controls.** The y-axis represents the quantitative data of the relative mRNA expression of *KL* and *TNF*. The x-axis corresponds to the two groups: *post mortem* controls (n = 5, circles) and TLE(HS) patients (n = 14, squares and triangles). In the control group, the unfilled circle represents the calibrator sample, whose gene expression is always 1.0. The combination of *ENO2* and *TBP* was used as the reference gene. Mann–Whitney U tests were used for comparison between groups. TLE(HS), temporal lobe epilepsy associated with hippocampal sclerosis.

Our results (Figure 1) showed a dramatic increase on *TNF* relative mRNA expression in hippocampal tissue of TLE(HS) patients in comparison with the *post mortem* controls (*P* <0.005, regardless of the reference gene). Despite the same *P* value for the results regarding the four reference genes used, the *TNF* expression mean in patients had an ample variation: from 29.61 ± 8.55 (*TBP*) to 110.4 ± 58.82 (*HPRT1*). The combination of *ENO2* and *TBP* as the normalization factor proved to be very stable in epileptogenic tissues [18]. Therefore, in Figure 2, we used this combination to show the relative *TNF* expression individually by subject.

Our data suggests that the marked *TNF* upregulation in patients’ tissues corroborates with the chronic hippocampal inflammation in TLE(HS). Table 1 shows that several patients had their last seizure several days before the surgery, suggesting that the high *TNF* expression levels were frequent, signaling that chronic hippocampal inflammation could be intrinsic to the refractory TLE(HS) syndrome. A number of studies in animal models indicate that seizures induce *TNF* expression in the brain [9,11-14]. Our findings confirm that a similar induction also occurs in TLE patients. However, despite the intense investigation of the TNF system and its effects in seizure models, there is not unanimous agreement on its effects in TLE(HS) and clinical studies are scarce [11]. Moreover, similar to recent findings on inflammatory disease models [16,17], our data indicates that the synthesis of KL is reduced in the sclerotic hippocampus and TNF may downregulate KL through NFkB in TLE(HS) (Figures 1 and 2).

While *KL* mRNA was significantly downregulated (*HPRT1* and *TBP*: *P* <0.001; *GAPDH*: *P* <0.02; *ENO2*: *P* <0.03), the expression level of *NFKB1* was also augmented in patients (*GAPDH*, *HPRT1* and *ENO2*: *P* <0.02; *TBP*: *P* <0.03) (Figure 1). It remains to be elucidated whether the KL depressed expression has a role in TLE(HS) physiopathology or is only a secondary change caused by the imbalance of calcium or phosphate and/or the progression of HS in the medial TLE syndrome. Since KL is a pleiotropic protein with different functions on many systems, our findings on *KL* downregulation opens up a gamut of study possibilities in HS pathophysiology comprehension.

We initially highlighted two specific functions assigned to KL: it has a stimulating effect on the Na^+^/K^+^ ATPase pump activity [22] and it influences the Wnt signal pathway [23]. Considering the importance of Na^+^/K^+^ ATPase activity in neuronal electrical activity and excitability [24], it is important to determine if KL regulates Na^+^/K^+^ ATPase in the hippocampus, which could be involved with the evident synaptic reduction found in *Kl*^*−/−*^ mice [6,7]. The molecular mechanisms regarding the neurodegenerative feature found in *Kl*^*−/−*^ mice and analogously to HS have not yet been elucidated. By acting on the Wnt signaling, KL offers an interesting outlook as it suppresses several Wnt family members [23]. It has been demonstrated that stimulation of Wnt signaling contributes to stem and progenitor cell senescence, and persistent decreased KL expression may affect the rate of cellular aging and have harmful impact on tissue repair mechanisms [17]. This could be a key factor in HS progression.

There is another connection between KL depression and astrogliosis. By suppressing TP53 and negatively regulating cyclin-dependent kinase inhibitor 1 (CDKN1A, also known as p21) protein levels, KL not only inhibits apoptosis but also modulates the lifespan of human cells, which may be associated with increased signaling through the insulin/insulin-like growth factor 1 (IGF-1) pathway [8]. This growth factor has a significant role in non-neuronal modulation and multiple reports have indicated that astrocytes are the main target of IGF-1 by regulating their response to tissue injury [25]. Since KL may inhibit the insulin/IGF-1 pathway [26], its downregulation could support astrogliosis by a detrimental effect on neurons and astrocyte proliferation. In fact, *Kl*^*−/−*^ mice have neuronal cell degeneration with a drastic increase in GFAP levels [27]. As expected, *GFAP* was upregulated in patients (*GAPDH*, *HPRT1* and *ENO2*: *P* <0.001; *TBP*: *P* <0.02), which supports astrogliosis (Figure 1).

Furthermore, KL has been considered an anti-inflammatory protein and this property could be one of its most striking features. In the endothelium, KL confers protection against nitric oxide (NO)-induced dysfunction [28], reduces the expression of adhesion molecules [28] and potentially regulates T cell functions [29]. In 2007, Witkowski *et al.* reported that KL was downregulated in CD4^+^ lymphocytes at the mRNA, protein and enzymatic (beta-glucoronidase) activity levels in healthy, older adults and especially in rheumatoid arthritis (RA) patients [29]. Regardless of the unclear link between KL activity and CD4^+^ cell function, they proposed that KL might be involved in physiological anti-inflammatory responses in young individuals, but these responses decreased in both healthy older adults and RA patients. Their hypothesis was supported by the fact that KL expression and activity reduction, in both older adults and RA patients’ lymphocytes, occurred in concert with the downregulation of CD28, a TNF-increased dependent T cell costimulatory molecule.

The *Kl*^*−/−*^ mouse also exhibits atherosclerosis and endothelial dysfunction, which led Maekawa *et al*. [28] to test the effect of KL on vascular inflammation. KL suppressed the TNF-induced expression of adhesion molecules and NFkB activation in endothelial cells *in vitro* (human umbilical vein endothelial cells (HUVECs)) and *ex vivo* (organ culture of the rat aorta). Moreover, KL reversed the repression of the nitric oxide synthase 3 (NOS3) phosphorylation by TNF and inhibited the TNF-induced monocyte adhesion to HUVECs. These results suggest that KL may have a function in the modulation of endothelial inflammation, especially by TNF-induced NFkB inhibition. Therefore, it is plausible that KL downregulation could further exacerbate the TLE(HS) associated chronic inflammatory condition.

Moreno *et al*. related that the inflammatory cytokines tumor necrosis factor ligand superfamily member 12 (TNFSF12, also known as TWEAK) and TNF downregulate KL in renal tubular cells through an NFkB-dependent mechanism mediated by histone deacetilase 1 (HDAC1) [16]. In this regard, they observed that the HDAC inhibitors trichostatin A (TSA) or valproic acid prevented repression of KL induced by TWEAK or TNF. Among the patients studied, only TLE 09 was taking valproate during the surgery and only TLE 06 took this medication in the past. We did not observe any particular difference in these patients compared with the others. *KL* expression was negatively correlated with *NFBK1* or *TNF* expression, while the pair *TNF* and *NFKB1* showed a positive correlation. This suggests that NFkB in patients is most likely modulated by TNF or even KL [28] and not by AEDs, although further studies are required to test the influence of AEDs in the gene expression of our targets. Gene expression data correlation was found in the three pairs tested: *KL*-*NFKB1* (Spearman’s R = −0.3140; *P* = 0.0091), *KL*-*TNF* (Spearman’s R = −0.3283; *P* = 0.0063), and *NFKB1*-*TNF* (Spearman’s R = 0.4441; *P* = 0.0001).

In addition, Thurston *et al.* showed that in mouse models of inflammatory bowel disease the degree of KL inhibition was related to the severity of colitis and that attenuation of inflammation with a neutralizing anti-TNF antibody impeded this inhibition [17]. Furthermore, the neurodegenerative feature found in *Kl*^*−/−*^ mice suggests that KL in the hippocampus may act as a protective autocrine hormone and its absence causes neuronal loss [30].

Our work on the mRNA level suggests that the triad *KL-NFKB1-TNF* is disrupted in the hippocampus from medically intractable TLE patients. Based on the KL function studies discussed here, we propose the first mechanistic insights into the role that this triad may play in the pathogenesis of medial TLE. It is conceivable that there is a major involvement of the KL-TNF axis in the pathogenesis of TLE(HS), particularly under chronic inflammatory conditions. Further research on the protein level will strengthen our results and the design of functional studies will be able to elucidate the role of our targets, especially KL, in the normal and pathological hippocampus. Due to the KL hormonal property [7,31] and TNF tissue diffusion as a cytokine [32,33], we believe that the epilepsy-associated inflammation is a widespread event in the hippocampus. Finally, since KL is detectable in cerebrospinal fluid [7,31,34], it is a potential candidate as an inflammatory biomarker in epilepsy. The inflammatory component of epilepsy is not a secondary phenomenon or complication of the pathology. It is more likely involved in the mechanisms that sustain neuronal hyperexcitability, the onset and recurrence of seizures, and progression and severity of the disease [15]. The determination of a reliable biomarker of brain inflammation is urgent, in view of the fact that a number of patients would benefit from an anti-inflammatory therapy.

## Abbreviations

AED: antiepileptic drug; CBZ: carbamazepine; CDKN1A: cyclin-dependent kinase inhibitor 1 (p21 Cip1); DEPC: diethylpyrocarbonate; EEG: electroencephalogram; ENO2: enolase 2 (gamma neuronal); GAPDH: glyceraldehyde 3-phosphate dehydrogenase; GFAP: glial fibrillary acidic protein; HDAC1: histone deacetilase 1; HGNC: HUGO Gene Nomenclature Committee; HPRT1: hypoxanthine phosphoribosyltransferase 1; HS: hippocampal sclerosis; HUVEC: human umbilical vein endothelial cell; IGF-1: insulin-like growth factor 1 (somatomedin C); IML: Instituto Médico Legal; KL: Klotho; Kl−/−: Klotho knockout; LTG: lamotrigine; MRI: magnetic resonance imaging; NFkB: nuclear factor kappa B; NFKB1: nuclear factor of kappa light polypeptide gene enhancer in B-cells 1; NOS3: nitric oxide synthase 3 (endothelial cell); OXC: oxcarbazepine; PCR: polymerase chain reaction; PHT: phenytoin; RA: rheumatoid arthritis; RIN: RNA integrity number; RT-qPCR: reverse transcription quantitative PCR; TBP: TATA box binding protein; TLE: temporal lobe epilepsy; TLE(HS): temporal lobe epilepsy associated with hippocampal sclerosis; TNF: tumor necrosis factor; TNFSF12: tumor necrosis factor ligand superfamily member 12 (TWEAK); TP53: tumor protein p53; TSA: trichostatin A; VPA: valproate.

## Competing interest

All authors declare that they have no competing interest.

## Authors’ contributions

MAT designed the study, collected clinical data, performed the experiments, analyzed the data, prepared the figures and tables, and drafted the manuscript. AEDF collected clinical data and helped to perform the experiments. EPLO and HT are neurosurgeons and operated on patients. LD-L designed and coordinated the study and helped to draft the manuscript. All authors read and approved the final manuscript.

## References

[B1] Semah F Picot MC Adam C Broglin D Arzimanoglou A Bazin B Cavalcanti D Baulac M Is the underlying cause of epilepsy a major prognostic factor for recurrence? Neurology 1998 51 1256 1262 10.1212/WNL.51.5.1256 9818842

[B2] Greenfield JG Graham DI Lantos PL Greenfield’s Neuropathology 1997 6 London: Arnold

[B3] Delorenzo RJ Sun DA Deshpande LS Cellular mechanisms underlying acquired epilepsy: the calcium hypothesis of the induction and maintainance of epilepsy Pharmacol Ther 2005 105 229 266 10.1016/j.pharmthera.2004.10.004 15737406 PMC2819430

[B4] Raza M Deshpande LS Blair RE Carter DS Sombati S DeLorenzo RJ Aging is associated with elevated intracellular calcium levels and altered calcium homeostatic mechanisms in hippocampal neurons Neurosci Lett 2007 418 77 81 10.1016/j.neulet.2007.03.005 17374449 PMC2094130

[B5] Huang CL Moe OW Klotho: a novel regulator of calcium and phosphorus homeostasis Pflugers Arch 2011 462 185 193 10.1007/s00424-011-0950-5 21442474

[B6] Kuro-o M Matsumura Y Aizawa H Kawaguchi H Suga T Utsugi T Ohyama Y Kurabayashi M Kaname T Kume E Iwasaki H Iida A Shiraki-Iida T Nishikawa S Nagai R Nabeshima YI Mutation of the mouse klotho gene leads to a syndrome resembling ageing Nature 1997 390 45 51 10.1038/36285 9363890

[B7] Li SA Watanabe M Yamada H Nagai A Kinuta M Takei K Immunohistochemical localization of Klotho protein in brain, kidney, and reproductive organs of mice Cell Struct Funct 2004 29 91 99 10.1247/csf.29.91 15665504

[B8] de Oliveira RM Klotho RNAi induces premature senescence of human cells via a p53/p21 dependent pathway FEBS Lett 2006 580 5753 5758 10.1016/j.febslet.2006.09.036 17014852

[B9] Ravizza T Balosso S Vezzani A Inflammation and prevention of epileptogenesis Neurosci Lett 2011 497 223 230 10.1016/j.neulet.2011.02.040 21362451

[B10] Foresti ML Arisi GM Shapiro LA Role of glia in epilepsy-associated neuropathology, neuroinflammation and neurogenesis Brain Res Rev 2011 66 115 122 10.1016/j.brainresrev.2010.09.002 20837059

[B11] Li G Bauer S Nowak M Norwood B Tackenberg B Rosenow F Knake S Oertel WH Hamer HM Cytokines and epilepsy Seizure 2011 20 249 256 10.1016/j.seizure.2010.12.005 21216630

[B12] Plata-Salaman CR Ilyin SE Turrin NP Gayle D Flynn MC Romanovitch AE Kelly ME Bureau Y Anisman H McIntyre DC Kindling modulates the IL-1beta system, TNF-alpha, TGF-beta1, and neuropeptide mRNAs in specific brain regions Brain Res Mol Brain Res 2000 75 248 258 10.1016/S0169-328X(99)00306-X 10686345

[B13] Godlevsky LS Shandra AA Oleinik AA Vastyanov RS Kostyushov VV Timchishin OL TNF-alpha in cerebral cortex and cerebellum is affected by amygdalar kindling but not by stimulation of cerebellum Pol J Pharmacol 2002 54 655 660 12866721

[B14] Vezzani A Moneta D Richichi C Aliprandi M Burrows SJ Ravizza T Perego C De Simoni MG Functional role of inflammatory cytokines and antiinflammatory molecules in seizures and epileptogenesis Epilepsia 2002 43 Suppl 5 30 35 12121291 10.1046/j.1528-1157.43.s.5.14.x

[B15] Vezzani A Friedman A Brain inflammation as a biomarker in epilepsy Biomark Med 2011 5 607 614 10.2217/bmm.11.61 22003909 PMC3625731

[B16] Moreno JA Izquierdo MC Sanchez-Nino MD Suarez-Alvarez B Lopez-Larrea C Jakubowski A Blanco J Ramirez R Selgas R Ruiz-Ortega M Egido J Ortiz A Sanz AB The inflammatory cytokines TWEAK and TNF-alpha reduce renal Klotho expression through NF-kappaB J Am Soc of Nephrol 2011 22 1315 1325 10.1681/ASN.2010101073 21719790 PMC3137579

[B17] Thurston RD Larmonier CB Majewski PM Ramalingam R Midura-Kiela M Laubitz D Vandewalle A Besselsen DG Muhlbauer M Jobin C Kiela PR Ghishan FK Tumor necrosis factor and interferon-gamma down-regulate Klotho in mice with colitis Gastroenterology 2010 138 1384 1394 10.1053/j.gastro.2009.12.002 20004202 PMC3454518

[B18] Wierschke S Gigout S Horn P Lehmann TN Dehnicke C Brauer AU Deisz RA Evaluating reference genes to normalize gene expression in human epileptogenic brain tissues Biochem Biophys Res Commun 2010 403 385 390 10.1016/j.bbrc.2010.10.138 21081112

[B19] Becker AJ Chen J Paus S Normann S Beck H Elger CE Wiestler OD Blumcke I Transcriptional profiling in human epilepsy: expression array and single cell real-time qRT-PCR analysis reveal distinct cellular gene regulation Neuroreport 2002 13 1327 1333 10.1097/00001756-200207190-00023 12151797

[B20] Ozbas-Gerceker F Redeker S Boer K Ozguc M Saygi S Dalkara T Soylemezoglu F Akalan N Baayen JC Gorter JA Aronica E Serial analysis of gene expression in the hippocampus of patients with mesial temporal lobe epilepsy Neuroscience 2006 138 457 474 10.1016/j.neuroscience.2005.11.043 16413123

[B21] Durrenberger PF Fernando S Kashefi SN Ferrer I Hauw JJ Seilhean D Smith C Walker R Al-Sarraj S Troakes C Palkovits M Kasztner M Huitinga I Arzberger T Dexter DT Kretzschmar H Reynolds R Effects of antemortem and postmortem variables on human brain mRNA quality: a BrainNet Europe study J Neuropathol Exp Neurol 2010 69 70 81 10.1097/NEN.0b013e3181c7e32f 20010301

[B22] Sopjani M Alesutan I Dermaku-Sopjani M Gu S Zelenak C Munoz C Velic A Foller M Rosenblatt KP Kuro-o M Lang F Regulation of the Na+/K+ ATPase by Klotho FEBS Lett 2011 585 1759 1764 10.1016/j.febslet.2011.05.021 21605558

[B23] Liu H Fergusson MM Castilho RM Liu J Cao L Chen J Malide D Rovira II Schimel D Kuo CJ Gutkind JS Hwang PM Finkel T Augmented Wnt signaling in a mammalian model of accelerated aging Science 2007 317 803 806 10.1126/science.1143578 17690294

[B24] Brines ML Tabuteau H Sundaresan S Kim J Spencer DD de Lanerolle N Regional distributions of hippocampal Na+, K(+)-ATPase, cytochrome oxidase, and total protein in temporal lobe epilepsy Epilepsia 1995 36 371 383 10.1111/j.1528-1157.1995.tb01012.x 7607116

[B25] Fernandez S Fernandez AM Lopez-Lopez C Torres-Aleman I Emerging roles of insulin-like growth factor-I in the adult brain Growth Horm IGF Res 2007 17 89 95 10.1016/j.ghir.2007.01.006 17317256

[B26] Wang Y Sun Z Current understanding of klotho Ageing Res Rev 2009 8 43 51 10.1016/j.arr.2008.10.002 19022406 PMC2637560

[B27] Shiozaki M Yoshimura K Shibata M Koike M Matsuura N Uchiyama Y Gotow T Morphological and biochemical signs of age-related neurodegenerative changes in klotho mutant mice Neuroscience 2008 152 924 941 10.1016/j.neuroscience.2008.01.032 18343589

[B28] Maekawa Y Ishikawa K Yasuda O Oguro R Hanasaki H Kida I Takemura Y Ohishi M Katsuya T Rakugi H Klotho suppresses TNF-alpha-induced expression of adhesion molecules in the endothelium and attenuates NF-kappaB activation Endocrine 2009 35 341 346 10.1007/s12020-009-9181-3 19367378

[B29] Witkowski JM Soroczynska-Cybula M Bryl E Smolenska Z Jozwik A Klotho–a common link in physiological and rheumatoid arthritis-related aging of human CD4+ lymphocytes J Immunol 2007 178 771 777 17202338 10.4049/jimmunol.178.2.771

[B30] Abraham CR Chen C Cuny GD Glicksman MA Zeldich E Small-molecule Klotho enhancers as novel treatment of neurodegeneration Future Med Chem 2012 4 1671 1679 10.4155/fmc.12.134 22924505 PMC3564652

[B31] German DC Khobahy I Pastor J Kuro OM Liu X Nuclear localization of Klotho in brain: an anti-aging protein Neurobiol Aging 2012 33 1483 22245317 10.1016/j.neurobiolaging.2011.12.018 PMC3328593

[B32] Falsig J Porzgen P Lotharius J Leist M Specific modulation of astrocyte inflammation by inhibition of mixed lineage kinases with CEP-1347 J Immunol 2004 173 2762 2770 15294995 10.4049/jimmunol.173.4.2762

[B33] Nadeau S Rivest S Role of microglial-derived tumor necrosis factor in mediating CD14 transcription and nuclear factor kappa B activity in the brain during endotoxemia J Neurosci 2000 20 3456 3468 10777809 10.1523/JNEUROSCI.20-09-03456.2000 PMC6773126

[B34] Sathyanesan M Girgenti MJ Banasr M Stone K Bruce C Guilchicek E Wilczak-Havill K Nairn A Williams K Sass S Duman JG Newton SS A molecular characterization of the choroid plexus and stress-induced gene regulation Transl Psychiatry 2012 2 e139 10.1038/tp.2012.64 22781172 PMC3410626

